# Learning to explore tree neighbourhoods for phylogenetic inference

**DOI:** 10.1093/bib/bbaf732

**Published:** 2026-01-19

**Authors:** Federico Julian Camerota Verdù, Andrea Gasparin, Luca Bortolussi, Lorenzo Castelli

**Affiliations:** Department of Engineering and Architecture, University of Trieste, Via Alfonso Valerio 6/1, 34127 Trieste, Italy; Department of Mathematics, Informatics and Geoscience, University of Trieste, Via Weiss 2, 34128 Trieste, Italy; Department of Engineering and Architecture, University of Trieste, Via Alfonso Valerio 6/1, 34127 Trieste, Italy; Department of Mathematics, Informatics and Geoscience, University of Trieste, Via Weiss 2, 34128 Trieste, Italy; Department of Engineering and Architecture, University of Trieste, Via Alfonso Valerio 6/1, 34127 Trieste, Italy

**Keywords:** phylogenetics, balanced minimum evolution, reinforcement learning, online adaptation, local search

## Abstract

Phylogenetic inference is a key challenge in computational biology, with applications ranging from evolutionary analysis to comparative genomics. The balanced minimum evolution problem (BMEP) offers a well-established formulation of this problem, but remains computationally intractable for large instances. In this work, we propose a reinforcement learning (RL) framework to tackle the BMEP through local search in the space of phylogenetic trees. Our contributions are three-fold: (i) we introduce an improved RL formulation tailored to the structure of phylogenetic inference in the context of the BMEP; (ii) we train an RL agent capable of solving instances with up to 100 taxa; and (iii) we investigate the generalization capabilities of the learned policy across different substitution models, instance sizes, and datasets. To address the limitations of relying solely on the learned policy at inference time, we integrate it into a novel search-based framework that enables effective adaptation during evaluation. Experimental results show that our method outperforms greedy heuristics and matches the performance of state-of-the-art algorithms for the BMEP. When tested under significant distributional shifts, we greatly reduce the gap with state-of-the-art algorithms. This demonstrates the potential of RL applications to phylogenetic inference.

## Introduction

Phylogenetics investigates evolutionary relationships among organisms, aiming to reconstruct their ancestral origins and divergence pathways. By analysing molecular data, such as DNA and protein sequences, researchers can infer these relationships in the form of a phylogenetic tree. The task of phylogenetic inference involves estimating both the topology and the branch lengths of a tree, with branch lengths reflecting evolutionary distances. When inference relies solely on molecular data, the resulting trees are often assumed binary and, in most cases, *unrooted*, i.e. lacking a specified ancestral root. Several approaches exist for estimating phylogenies from molecular data, including clustering algorithms and optimization-based methods. In particular, distance-based approaches offer a computationally efficient alternative by constructing phylogenetic trees from pairwise dissimilarity values, i.e. the estimated evolutionary distance between distinct aligned molecular sequences (such as DNA, RNA, or codon sequences), hereafter referred to as *taxa*. For example, the Neighbour-Joining algorithm [1] infers tree topologies by iteratively joining the pair of taxa with the smallest evolutionary distance.

This work focuses on distance-based phylogenetic inference under the minimum evolution (ME) criterion that adopts a parsimony-inspired principle, hypothesizing that the most plausible phylogenetic tree is the one with the smallest total branch length [2, 3]. In particular, the balanced minimum evolution problem (BMEP) is a refined model grounded in the ME principle which guarantees a set of robust statistical properties [4]. The BMEP formulates phylogenetic inference as a discrete, nonlinear, and non-convex optimization problem over unrooted binary trees [5, 6], aiming to minimize total branch length. Given a set of $N$ taxa, the BMEP consists of finding the phylogeny $T$ that minimizes the length function


\begin{align*} &\mathcal{L}(T) = \sum_{i, j | i \neq j} \frac{d_{ij}}{2^{\tau_{ij}}}\end{align*}

where $d_{ij}$ is the estimated evolutionary distance between taxa $i$ and $j$ and $\tau _{ij}$ is the path length, i.e. the number of edges in the path between taxa $i$ and $j$ in the phylogeny $T$. BMEP builds on the branch length estimation method [7]that enables efficient approximation and makes the approach suitable for large-scale datasets. This method was further extended into a broader framework [8] that has since been refined and analysed in subsequent studies [4]. The theoretical properties of BMEP have attracted significant attention, particularly in the analysis of its combinatorial structure and optimization landscape [5, 6, 9–12]. Although it was shown that the BMEP can be solved in polynomial time for special kinds of input matrices [13], real-world data rarely belong to these categories, so the problem remains, in most cases, hard to solve. In particular, a major result demonstrated that BMEP is in general NP-hard [14], implying that no polynomial-time algorithm exists for solving it in general unless $\mathrm{P} = \mathrm{NP}$ [15]. Further complicating the picture, it was proved that the Fixed-Tree BMEP, a simpler variant in which the tree topology is fixed and only the taxon assignment is optimized, remains NP-hard [16]. The complexity of solving the BMEP has significantly constrained the success of exact algorithms. Despite advances in computational methods, exact approaches struggle with scalability as the number of taxa increases. Implicit enumeration [17–19] and integer programming methods [5] have been effective for small instances but remain limited to problems with at most 26 taxa. This highlights the exponential growth of the solution space and the intractability of searching for exact solutions of larger datasets.

Given these limitations, heuristic algorithms have been developed to provide efficient approximations. While not guaranteeing optimality, heuristics enable the exploration of large solution spaces without exhaustive searches. Notable contributions have improved the scalability and accuracy of phylogenetic inference under the BMEP framework [8, 14, 17, 20]. Among them, FastME $2.0$ [21] stands out for its speed, accuracy, and ability to handle large molecular datasets. Starting from an initial tree, FastME iteratively performs the topological rearrangement (or moves) that at each step minimizes the total branch length [13], until no move can provide further improvements. The algorithm has been proven to be particularly effective when moves are selected among the set of subtree pruning and regrafting (SPR) moves [22], which is the set of all possible rearrangements obtainable by detaching a subtree and reinserting it elsewhere. However, such a greedy strategy can lead to premature convergence to local optima, particularly in complex evolutionary scenarios. In addition, the solution quality is highly dependent on the initial tree [23]. To address these limitations PhyloES was introduced [24], a heuristic that integrates evolution strategies principles [25] to enhance search diversity, and is currently the state-of-the-art for the BMEP. PhyloES builds upon FastME’s local search but introduces stochastic recombination operators that expand the exploration of the solution space. It alternates between local search and recombination phases, balancing the exploitation of the greedy algorithm and the exploration of alternative tree structures. This iterative approach increases the likelihood of obtaining high-quality solutions, especially for complex phylogenies. Still, the local search is performed on a greedy basis, and one might wonder if different SPR moves selection policies might lead to better results.

In recent years, machine learning (ML) has gained traction in biological research [26–29], including in phylogenetic inference. Early ML-based methods focused on supervised learning, where models are trained to predict evolutionary relationships [27, 30–33]. While these approaches can be effective, they typically require large labelled datasets, which are often unavailable in real-world settings. More recently, reinforcement learning (RL) has emerged as a promising alternative, offering a more flexible and exploratory approach than traditional heuristics. RL methods learn decision policies that go beyond greedy selection by evaluating intermediate states and optimizing long-term outcomes [34–36]. This capability is particularly valuable in phylogenetic inference [36] where local improvements do not necessarily lead to globally optimal trees. These reasons make RL a promising research direction to improve current state-of-the-art algorithms such as PyloES or FastME, since their main limitation is that they both heavily rely on greedy heuristics. However, early RL-based methods for phylogenetic inference still struggled with scalability and generalization, i.e. the ability to perform well on problems significantly different to the ones used for their training. The latter is of particular interest as it is still a well-known open challenge for RL-based combinatorial optimization heuristics [37]. Initial efforts targeted both distance-based [34, 35] and maximum-likelihood [36] frameworks, but none performed effectively on problems involving more than $20$ taxa. In particular, Azouri *et al.* [36] assess the performance of their model when applied to smaller instances than those it was trained on, but scalability and generalization are not considered; thus, the performance of the proposed model when applied to larger and considerably different datasets is not investigated. To address these limitations, in this work, we build upon recent advances in neural combinatorial optimization to propose an RL framework for the BMEP, which makes a significant step forward in terms of scalability and generalization. Our framework demonstrates, in fact, the ability to efficiently handle significantly larger problem instances (up to 100 taxa) compared to the aforementioned ML-based approaches, despite being trained exclusively on a limited set of smaller-scale problems (30 taxa). Furthermore, it exhibits strong performance even under substitution models not encountered during training, highlighting its robust generalization capabilities. To build our framework, we leverage the well-defined combinatorial structure of the BMEP and its theoretically grounded operators. Specifically, we use SPR moves to train an agent that explores the trees’ space, with the goal of improving upon the standard greedy strategies in current state-of-the-art heuristics. We then incorporate an online search and adaptation mechanism, which fine-tunes model parameters to the specific problem instance being solved, and we demonstrate how this method plays a crucial role in enhancing model generalization. Extensive experiments show that our approach matches the state-of-the-art methods on problems with the same size and substitution model as the ones used for training, while preserving satisfactory performance on larger and more diverse instances. Our work thus provides a foundation for overcoming the critical challenges of scaling and generalization, which are fundamental to applying ML effectively to real-world phylogenetic problems.

## Materials and methods

### Reinforcement learning

RL provides a powerful mathematical framework for sequential decision-making problems, where an agent learns to maximize cumulative rewards through direct interaction with its environment. Unlike supervised learning, which requires labelled examples, or unsupervised learning that discovers hidden patterns, RL enables agents to learn optimal strategies through trial-and-error, guided only by feedback in the form of rewards. This paradigm has proven particularly effective in domains requiring adaptive decision-making under uncertainty, including robotics [38], game playing [39], and combinatorial optimization [40].

The RL framework is formally grounded in the theory of *Markov Decision Processes* (MDPs) [41]. An MDP is defined by the tuple $(\mathcal{S}, \mathcal{A}, P, r, \gamma )$, where $\mathcal{S}$ represents the state space, encompassing all possible configurations of the environment; $\mathcal{A}$ denotes the action space containing all available decisions; $P(s^{\prime}|s,a)$ specifies the transition dynamics governing state evolution from $s$ to $s^{\prime}$ given action $a$; $r: \mathcal{S} \times \mathcal{A} \rightarrow \mathbb{R}$ is the reward function providing immediate feedback to the agent; and $\gamma \in [0,1]$ is the discount factor balancing immediate versus future rewards.

At each timestep $t$, the agent observes the current state $S_{t}$, selects an action $A_{t}$ according to its *policy*  $\pi $, then receives a reward $R_{t}$, and the environment transitions to a new state $S_{t+1}$. The policy $\pi : \mathcal{S} \rightarrow \mathcal{P}(\mathcal{A})$ maps states to probability distributions over actions and may be either deterministic or stochastic. The stochastic formulation:


(1) \begin{align*}& \pi(a|s) = P(A_{t} = a | S_{t} = s)\end{align*}

allows for exploration during learning. The objective is to find an optimal policy $\pi ^{*}$ that maximizes the *expected return*:


(2) \begin{align*}& G_{t} = \mathbb{E}_{\pi^{*}}\left[\sum_{k=0}^{T} \gamma^{k} R_{t+k}\right]\end{align*}

where $T$ is the horizon of the agent-environment interaction (also called *episode*).

### Philogenetic inference as a reinforcement learning problem

**Figure 1:**
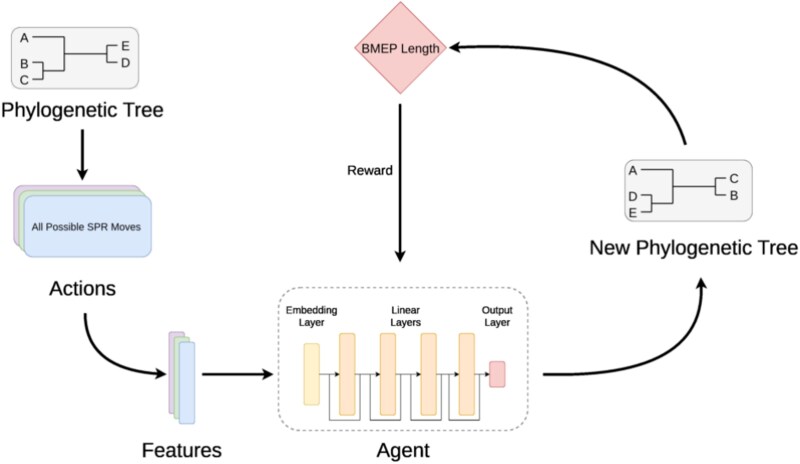
Flowchart representation of the RL framework employed for phylogenetic tree reconstruction.

A schematic overview of our RL framework is provided in Fig. 1, which depicts the components used to train neural agents for phylogenetic tree reconstruction. To tackle the phylogenetic inference problem, given a BMEP instance, an initial random solution is built. The RL agent is then trained to explore the solution space by selecting SPR moves that improve the starting phylogeny over the course of several steps. To cast phylogenetic inference as an MDP, we defined: (i) the state space $\mathcal{S}$ as the set of all possible unrooted tree topologies for a given set of taxa; (ii) the action space $\mathcal{A}(s)$ as the set of all SPR moves applicable to the current tree topology $\delta $; and (iii) the reward function $r(s, s^{\prime})$ as a measure of quality improvement when transitioning from state $s$ to $s^{\prime}$ via an SPR move.

For the BMEP, we defined the reward function using tree lengths and introduce the best-known solution $\delta ^{*}$ as an additional state component:


(3) \begin{align*}& r(s, s^{\prime}) = \max\left(\frac{L_{\delta^{*}} - L_{\delta^{\prime}}}{L_{\delta^{0}}}, 0\right)\end{align*}

where $\delta ^{0}$ is a randomly initialized tree sampled at the beginning of each episode and $\delta ^{\prime}$ is the tree obtained after the SPR move. This reward design encourages the agent to pursue global improvements by considering only improvements over the current best solution $\delta ^{*}$. Indeed, contrary to other approaches, that tend to favour immediate improvements over the current solution, such as in that of Azouri *et al.* [36] (which used a Deep Q-Network [42]), our reward tries to avoid the policy from cycling between slightly better and worse solutions, a common issue in step-wise reward designs [43]. In fact, without this precaution, a policy that simply learns to cycle between two fixed solutions would be rewarded every time it transitions from the worst to the better one, achieving a large cumulative reward, which might lead the RL algorithm to favour policies that do not solve the problem. Thus, the reward signal in this case is not aligned with the task we want the model to learn. Instead, with our framework, only improvements from the best found tree are rewarded, regardless of whether they come after several worsening steps, favouring stronger results over a long-term horizon rather than short-term gains. Although this design introduces a sparse reward signal, since only improving actions are rewarded, it did not hinder training in our experiments. In principle, however, such sparsity could make policy optimization more challenging in settings where identifying improving moves is particularly difficult, e.g. in very large solution spaces. In our case, the exponential growth of the solution space with the number of taxa did not result in any observed degradation in learning performance. On the contrary, the substantial improvements obtained through online adaptation on the largest instances (100 taxa) indicate that the method remains effective despite the sparsity of the reward. Moreover, training the policy on smaller instances (30 taxa) helps mitigate potential issues related to sparse reward, as the denser reward signal at this scale supports stable and efficient learning.

For the action representation, we adapted to the BMEP the set of $19$ handcrafted features introduced by Azouri *et al.* [31] (Table 1) that describe each SPR move in terms of both the source tree $\delta $ and the resulting tree $\delta ^{\prime}$. These include metrics related to subtree topology and branch lengths, which in the BMEP case are estimated via the recursive formulas [8]. The estimated new branch length (feature 7 of Table 1) was computed via the recursive formulas [44]; this procedure also provides the length of $\delta ^{\prime}$. Since this information is directly correlated with the optimization objective, we included the length of $\delta ^{\prime}$ as an additional feature. This enriched representation enables the policy to make more informed decisions by considering both the structural and objective-specific properties of the trees.

**Table 1:** Features set used to represent SPR moves: $1$ to $7$ are computed on the initial tree, whereas from $8$ to $19$ are computed on the subtrees $a$, $b$, $b_{1}$, and $b_{2}$(the first two result from breaking the initial tree at the pruning branch, where $a$ represents the pruned subtree while $b_{1}$ and $b_{2}$ are the two subtrees obtained by breaking $b$ at the regrafting branch)

No. Feature	Feature name	Details	Tree considered
1	Total branch lengths	The sum of branch lengths in the starting tree	Initial tree
2	Longest branch	The length of the longest branch in the starting tree	
3–4	Branch length	The length of the branch that was being pruned or regrafted	
5	Topology distance from the pruned node	The number of branches in the path between the regrafting and pruning branches, not including these branches	
6	Branch length distance from the pruned node	The sum of branches in the path between the regrafting and pruning branches, not including these branches	
7	New branch length	The estimated length of the new branch formed due to pruning	
8–11	Number of species	The number of leaves in the four subtrees	Subtrees $a$, $b$, $b_{1}$, $b_{2}$
12–15	Total branch lengths	The sum of branch lengths in the four subtrees	
16–19	Longest branch	The length of the longest branch in the four subtrees	

### Learning to search in subtree pruning and regrafting neighbourhoods

In our approach, we tackled the BMEP by training a stochastic policy $\pi _\theta $ to navigate SPR neighbourhoods. The policy was implemented as a feed-forward neural network [45] and trained with the REINFORCE policy-gradient algorithm [46], which we selected for its simplicity and prior success in similar domains [47–49]. An initial embedding layer maps input features into a latent space of dimension $h = 128$, followed by four fully connected layers, each composed of $128$ neurons, with leaky ReLU activations and skip connections. The output layer produces a scalar score $l_\theta (x_{i})$ for each SPR move $x_{i}$, and a softmax transformation yields the action probabilities:


(4) \begin{align*}& \pi_\theta(x_{i}) = \frac{e^{l_\theta(x_{i})}}{\sum_{j} e^{l_\theta(x_{j})}}\end{align*}

While this architecture is quite simple, it turns out to be very effective in our experiments. Input features were standardized to improve training stability; in particular, features involving tree lengths were divided by the length of the current tree (excluding the tree’s own length), and count-based features were normalized by the total number of taxa. This ensured consistent feature scales across training instances.

A critical component of our framework is the use of an auxiliary sampling policy based on the observed improvement values ${\Delta _{i}}$ associated with each SPR move ${x_{i}}$. Instead of sampling directly from the learned policy, which made learning too hard in the early stages of training, we define a softmax distribution over improvements:


(5) \begin{align*}& \pi_{\mathrm{SPR}}(x_{i}) = \frac{e^{\Delta_{i}/\mu}}{\sum_{j} e^{\Delta_{j}/\mu}}\end{align*}

where $\mu $ is a softmax temperature parameter. High values of $\mu $ encourage random actions, while low values tend to favour actions based on the tree length improvement. To rely mainly on the greedy selection strategy but preserving a minimal level of exploration, we adopted a compromising approach, for which, after preliminary experiments, a good value turned out to be $\mu = 10^{-6}$. This hybrid strategy enabled the agent to exploit effective moves early in training while still exploring alternative actions. Over time, the learned policy performed better than this auxiliary distribution, indicating that the model has discovered more effective strategies than greedy improvement alone.

Building on this, we extended the scalability of the model by incorporating Limited Rollout Beam Search (LRBS) [50]. LRBS is an online search and adaptation approach developed for RL-based heuristics in neural combinatorial optimization that explores the search space with a *beam* of active solutions and performs *online* (OA) fine-tuning, i.e. model parameters are tuned to the instance being solved at inference time. This approach allowed scaling to larger instances, supporting a range of different substitution models, and generalizing to datasets not seen during training. Importantly, LRBS is model-agnostic, and its ability to improve generalization suggests that future advancements in RL-based heuristics for the BMEP can readily benefit from its integration. Moreover, the exploration and adaptation framework of LRBS is not tailored to our experimental setup, which makes it applicable also to other phylogenetic inference approaches.

## Experimental setup

Experiments were carried out on two benchmark datasets, *rdpii* and *zilla*, which have been widely used in earlier phylogenetic analyses [51, 52]. These datasets, available at https://github.com/stamatak/test-Datasets, include the first $200$ taxa from each source dataset. For our study, we splitted each dataset into training and validation subsets. Distance matrices for all experiments were computed using four well-established substitution models available in FastME: JC69 [53], K2P [54], F81 [55], and F84 [56]. The goal of our experiments was three-fold, as we aimed at investigating the capabilities of the RL model to: (i) scale on bigger instances with respect to the ones used for its training; (ii) generalize over different substitution models; and (iii) generalize across different datasets. To better address these research questions, we trained two models separately on $30$-taxa instances, one on the *zilla* dataset and one on the *rdpii* dataset, and exclusively on data generated using the F81 model. We note that to ensure optimal performance, one would typically need to train a model to solve instances of the same or similar sizes—fact that would make this approach highly impractical, as different problem sizes would require to train different models. Furthermore, incorporating multiple substitution models into a single dataset would likely produce a more powerful model, better adaptable to diverse scenarios. However, this choice would not align with the primary objective of our investigation. The decision to constrain the training dataset in terms of instance size and data variety is intentional, designed specifically to probe our framework’s ability to scale and generalize even when trained on limited and incomplete data.

### Baselines

We compared our method against PhyloES, which served as a reference point in all evaluations by providing tree lengths used to compute gaps for both our RL approach and other baselines. Hyper parameters of PhyloES have been set to the default values suggested by the authors (https://github.com/andygaspar/PHYLOES, [24]), with a population size of 16 trees, 1000 maximum iterations and a tolerance of $10^{-16}$ for the tree length comparison. In addition, we considered three baselines for comparison with the RL policy: (i) Greedy selection (*bspr*): a heuristic that selects SPR moves based on immediate improvement. (ii) Sampled SPR selection (*sample-bspr*): the $\pi _{\mathrm{SPR}}$ sampling policy defined by equation (5), which adds randomness to move selection. (iii) Greedy selection from multiple random starts (*fair-bspr*): a stronger baseline that runs the greedy policy from $10$ randomly initialized trees. This heuristic provides a fairer benchmark for the RL policy when used with LRBS, since both leverage the same number of multiple solutions.

### Training

The RL policy was trained over $200$ iterations, where each iteration involved solving a new batch of training problems consisting of $100$ instances, each featuring phylogenetic trees with $30$ taxa. To maintain diversity during training, the dataset was resampled every five iterations.

Policy updates have been performed according to the REINFORCE algorithm, a common practice in the neural combinatorial optimization literature [47–49]. To train the model, every $2$ iterations, $10$ epochs of policy training were performed over the accumulated experience. Each epoch was conducted with a batch size of $512$ samples. We used the Adam optimizer [57] with a learning rate of $0.001$ and a weight decay of $0.0001$ to mitigate overfitting. To evaluate validation performance, we monitored the agent on a fixed set of $20$ instances, sampled from a distinct pool of taxa separate from the training set. We saved model checkpoints whenever an improvement in validation performance was observed, allowing us to retain the most effective policy encountered during training. To obtain the models used for the assessment, the total training time required amounted to <3 h.

### Testing

During evaluation, we tested the RL policy on $100$ problem instances sampled from the complete datasets, with varying numbers of taxa. Although the RL agent was trained with a $30$-step improvement horizon, we increased this to $100$ steps at test time to enable more refined solutions. Initial experiments indicated that shorter horizons during training facilitated faster learning, whereas longer horizons during testing allowed the agent to make more incremental progress. In contrast, greedy baselines such as *bspr* and *fair-bspr* typically converged in fewer than $100$ steps due to their deterministic behaviour. This helped diversify the search process and avoid premature convergence to local optima. Finally, we note that while LRBS solves instances sequentially, other baseline methods can benefit from parallelism, suggesting that with suitable engineering, the efficiency of LRBS could be significantly improved. The selection of LRBS hyperparameters was conducted through preliminary analyses on a small set of representative instances. The choice ultimately followed two main criteria: first, achieving a favourable trade-off between solution quality and computational overhead, setting the search budget ($\alpha \times \beta $) to $10$; second, leveraging the findings from the original LRBS paper [50], where smaller $\alpha $ (or equivalently larger $\beta $) values performed best on instances comparable with those seen during training, while larger values were more effective on larger instances. For problem instances with $30$ and $70$ taxa, we configured the LRBS parameters $\alpha $ (the beam width) and $\beta $ (the explored children for each solution) as $\alpha = 10$, $\beta = 1$, while for larger instances with $100$ taxa, we adjusted the parameters to $\alpha = 2$, $\beta = 5$ to account for the increased complexity. During online adaptation, parameter updates were performed using the REINFORCE algorithm, with training data collected from the multiple LRBS beams while solving a single instance.

## Results

We now report the experimental results obtained by applying the framework introduced in Section “Learning to search in subtree pruning and regrafting neighbourhoods” to train a neural heuristic for the BMEP. Our findings demonstrate that employing an RL-driven policy for SPR move selection can outperform traditional greedy heuristics on the BMEP. Moreover, the approach yields performance comparable to state-of-the-art algorithms such as PhyloES. Notably, even when faced with significant distributional differences between training and testing data, the combination of the neural heuristic and online search methods sustains strong performance, underscoring our approach’s generalization capabilities and robustness. All experiments were executed using a single NVIDIA Ampere GPU equipped with $64$ GB of HBM2 memory and supported by $32$ CPU cores. All experiments and models are available at https://github.com/federico-camerota/bmepRL.

### Learning to search for the balanced minimum evolution problem

As a first test, we aimed at analysing the performance of our framework on instances similar (same size and substitution model) to the ones used during training. The first column in Table 2 displays the performance of our RL heuristic trained separately on both the *zilla* and *rdpii* datasets, each consisting of $30$-taxa instances. We initially observe that the *sample-bspr* baseline already shows a clear improvement over the deterministic *bspr* method. Such a result underscores the fact that injecting even mild randomness into greedy selection can yield better outcomes for the BMEP [23]. This highlights the potential of stochastic heuristics to enhance performance in phylogenetic inference tasks. Still, to demonstrate that the RL agent has learned more than just leveraging random variation, it must outperform the *sample-bspr* baseline. As shown in the first column of Table 2, this is the case, since the RL heuristic consistently achieves better results than both *bspr* and *sample-bspr*. For the *zilla* dataset, it nearly halves the gap relative to *sample-bspr*, substantially improving over simple greedy strategies. The improvements are even more significant on the *rdpii* dataset, where the reduction in the gap exceeds a factor of $2$, illustrating the RL agent’s effectiveness at solving BMEP instances.

**Table 2:** Performance evaluation (Obj: average objective value, Gap: average gap, and Time: total time) on *zilla* and *rdpii* datasets, using F81 substitution model, with $30$, $70,$ and $100$ taxa.

		$N = 30$			$N = 70$			$N = 100$		
	Method	Obj.	Gap (%)	Time	Obj.	Gap (%)	Time	Obj.	Gap (%)	Time
zilla	PhyloES	$0.718300$	$0.0000$	$0.2$ m	$1.426253$	$0.0000$	$1.0$ m	$1.886753$	$0.0000$	$2.4$ m
	bspr	$0.718460$	$0.0218$	$0.0$ m	$1.426994$	$0.0517$	$0.0$ m	$1.887820$	$0.0562$	$0.0$ m
	sample-bspr	$0.718415$	$0.0157$	$0.1$ m	$1.426922$	$0.0467$	$0.9$ m	$1.888625$	$0.0990$	$2.1$ m
	fair-bspr	$0.718301$	$0.0000$	$0.0$ m	$1.426303$	$0.0034$	$0.0$ m	$1.886883$	$0.0069$	$0.0$ m
	RL	$0.718360$	$0.0082$	$0.2$ m	$1.427685$	$0.0999$	$1.0$ m	$3.395346$	$80.096$	$1.8$ m
	RL+LRBS	$0.718301$	$0.0001$	$4.0$ m	$1.426534$	$0.0195$	$24.2$ m	$2.749682$	$45.376$	$53.5$ m
	RL+LRBS+OA	$0.718300$	$0.0000$	$8.0$ m	$1.426396$	$0.0097$	$42.2$ m	$1.890626$	$0.2047$	$85.9$ m
rdpii	PhyloES	$2.847522$	$0.0000$	$0.2$ m	$5.737708$	$0.0000$	$1.0$ m	$7.724478$	$0.0000$	$2.2$ m
	bspr	$2.847957$	$0.0153$	$0.0$ m	$5.739842$	$0.0372$	$0.0$ m	$7.726748$	$0.0294$	$0.0$ m
	sample-bspr	$2.847843$	$0.0113$	$0.1$ m	$5.739745$	$0.0354$	$0.9$ m	$7.728258$	$0.0490$	$2.1$ m
	fair-bspr	$2.847532$	$0.0003$	$0.0$ m	$5.737828$	$0.0021$	$0.0$ m	$7.724761$	$0.0036$	$0.0$ m
	RL	$2.847664$	$0.0050$	$0.2$ m	$7.030473$	$22.387$	$1.0$ m	$12.884346$	$66.862$	$1.8$ m
	RL+LRBS	$2.847522$	$0.0000$	$4.0$ m	$5.738179$	$0.0081$	$24.2$ m	$10.804071$	$39.901$	$52.8$ m
	RL+LRBS+OA	$2.847522$	$0.0000$	$8.0$ m	$5.738094$	$0.0067$	$42.0$ m	$7.780919$	$0.7265$	$86.1$ m

When compared with the *fair-bspr* baseline, *fair-bspr* already performs quite competitively, almost matching PhyloES in some cases. This highlights how much benefit can be gained from running greedy search from multiple initializations. However, the inclusion of LRBS with online adaptation yields even greater performance. On the *rdpii* dataset, for instance, the RL method combined with LRBS matches the PhyloES solution, marginally outperforming *fair-bspr*. These results confirm that online adaptation enables the RL heuristic to go beyond what is possible with purely greedy approaches, even those enhanced with randomized starting points.

### Scaling and generalization over substitution models

**Table 3:** Performance evaluation (Obj: average objective value, Gap: average gap, and Time: total time) on *zilla* and *rdpii* datasets, using F84 substitution model, with $30$, $70,$ and $100$ taxa.

		$N = 30$			$N = 70$			$N = 100$		
	Method	Obj.	Gap	Time	Obj.	Gap	Time	Obj.	Gap	Time
zilla	PhyloES	$0.717896$	$0.0000$	$0.2$ m	$1.420612$	$0.0000$	$1.0$ m	$1.907265$	$0.0000$	$2.7$ m
	bspr	$0.718080$	$0.0263$	$0.0$ m	$1.421511$	$0.0639$	$0.0$ m	$1.908666$	$0.0733$	$0.0$ m
	sample-bspr	$0.718059$	$0.0229$	$0.1$ m	$1.421411$	$0.0567$	$0.9$ m	$1.909539$	$0.1189$	$2.1$ m
	fair-bspr	$0.717896$	$0.0000$	$0.0$ m	$1.420655$	$0.0030$	$0.0$ m	$1.907460$	$0.0102$	$0.0$ m
	RL	$0.717982$	$0.0121$	$0.2$ m	$1.422001$	$0.0979$	$1.0$ m	$3.439934$	$80.545$	$1.8$ m
	RL+LRBS	$0.717896$	$0.0000$	$4.0$ m	$1.420853$	$0.0168$	$24.1$ m	$2.879633$	$50.581$	$53.3$ m
	RL+LRBS+OA	$0.717896$	$0.0000$	$8.0$ m	$1.420742$	$0.0091$	$42.2$ m	$1.911982$	$0.2461$	$86.0$ m
rdpii	PhyloES	$2.800681$	$0.0000$	$0.2$ m	$5.695594$	$0.0000$	$1.0$ m	$7.795419$	$0.0000$	$2.1$ m
	bspr	$2.801166$	$0.0177$	$0.0$ m	$5.697002$	$0.0247$	$0.0$ m	$7.798389$	$0.0378$	$0.0$ m
	sample-bspr	$2.801030$	$0.0127$	$0.1$ m	$5.696811$	$0.0213$	$0.9$ m	$7.800661$	$0.0672$	$2.1$ m
	fair-bspr	$2.800688$	$0.0002$	$0.0$ m	$5.695694$	$0.0017$	$0.0$ m	$7.795732$	$0.0040$	$0.0$ m
	RL	$2.800842$	$0.0058$	$0.2$ m	$6.731598$	$17.782$	$1.0$ m	$12.994180$	$66.789$	$1.8$ m
	RL+LRBS	$2.800681$	$0.0000$	$4.0$ m	$5.696047$	$0.0078$	$24.3$ m	$10.908665$	$39.990$	$52.8$ m
	RL+LRBS+OA	$2.800681$	$0.0000$	$8.0$ m	$5.695944$	$0.0061$	$42.3$ m	$7.853529$	$0.7423$	$86.2$ m

**Table 4:** Performance evaluation (Obj: average objective value, Gap: average gap, and Time: total time) on *zilla* and *rdpii* datasets, using JC69 substitution model, with $30$, $70,$ and $100$ taxa.

		$N = 30$			$N = 70$			$N = 100$		
	Method	Obj.	Gap	Time	Obj.	Gap	Time	Obj.	Gap	Time
zilla	PhyloES	$0.712853$	$0.0000$	$0.2$ m	$1.409966$	$0.0000$	$1.0$ m	$1.886958$	$0.0000$	$2.2$ m
	bspr	$0.713097$	$0.0342$	$0.0$ m	$1.410683$	$0.0504$	$0.0$ m	$1.888234$	$0.0675$	$0.0$ m
	sample-bspr	$0.713031$	$0.0248$	$0.1$ m	$1.410607$	$0.0451$	$0.9$ m	$1.888985$	$0.1071$	$2.1$ m
	fair-bspr	$0.712857$	$0.0005$	$0.0$ m	$1.410048$	$0.0057$	$0.0$ m	$1.887105$	$0.0077$	$0.0$ m
	RL	$0.712919$	$0.0094$	$0.2$ m	$1.411407$	$0.1020$	$1.0$ m	$3.388043$	$79.712$	$1.8$ m
	RL+LRBS	$0.712853$	$0.0000$	$4.1$ m	$1.410262$	$0.0207$	$24.1$ m	$2.721500$	$43.777$	$53.4$ m
	RL+LRBS+OA	$0.712853$	$0.0000$	$8.0$ m	$1.410097$	$0.0093$	$42.1$ m	$1.890830$	$0.2044$	$85.6$ m
rdpii	PhyloES	$2.825404$	$0.0000$	$0.2$ m	$5.668673$	$0.0000$	$0.9$ m	$7.689584$	$0.0000$	$2.0$ m
	bspr	$2.825930$	$0.0188$	$0.0$ m	$5.670186$	$0.0266$	$0.0$ m	$7.692278$	$0.0349$	$0.0$ m
	sample-bspr	$2.825774$	$0.0131$	$0.1$ m	$5.670109$	$0.0252$	$0.9$ m	$7.694217$	$0.0601$	$2.1$ m
	fair-bspr	$2.825411$	$0.0002$	$0.0$ m	$5.668740$	$0.0012$	$0.0$ m	$7.689865$	$0.0036$	$0.0$ m
	RL	$2.825677$	$0.0097$	$0.2$ m	$6.519664$	$14.672$	$1.0$ m	$12.900974$	$67.861$	$1.8$ m
	RL+LRBS	$2.825404$	$0.0000$	$4.0$ m	$5.669022$	$0.0061$	$24.3$ m	$10.784358$	$40.301$	$52.4$ m
	RL+LRBS+OA	$2.825404$	$0.0000$	$8.0$ m	$5.669033$	$0.0063$	$42.2$ m	$7.743175$	$0.6948$	$85.8$ m

To explore the boundaries of the RL agent’s capabilities to scale and generalize, we evaluated its performance on larger instances (up to $100$ taxa) and under various substitution models (F84, JC69, and K2P in addition to the F81 used for training). The results are shown in Tables 2–5.

**Table 5:** Performance evaluation (Obj: average objective value, Gap: average gap, and Time: total time) on *zilla* and *rdpii* datasets, using K2P substitution model, with $30$, $70,$ and $100$ taxa.

		$N = 30$			$N = 70$			$N = 100$		
	Method	Obj.	Gap	Time	Obj.	Gap	Time	Obj.	Gap	Time
zilla	PhyloES	$0.719698$	$0.0000$	$0.2$ m	$1.437240$	$0.0000$	$1.1$ m	$1.899995$	$0.0000$	$2.3$ m
	bspr	$0.719939$	$0.0326$	$0.0$ m	$1.438054$	$0.0570$	$0.0$ m	$1.901437$	$0.0752$	$0.0$ m
	sample-bspr	$0.719901$	$0.0276$	$0.1$ m	$1.437954$	$0.0499$	$0.9$ m	$1.902169$	$0.1138$	$2.1$ m
	fair-bspr	$0.719704$	$0.0008$	$0.0$ m	$1.437303$	$0.0043$	$0.0$ m	$1.900175$	$0.0094$	$0.0$ m
	RL	$0.719763$	$0.0089$	$0.2$ m	$1.438665$	$0.0993$	$1.0$ m	$3.422436$	$80.288$	$1.8$ m
	RL+LRBS	$0.719698$	$0.0000$	$4.0$ m	$1.437550$	$0.0215$	$24.1$ m	$2.837269$	$48.802$	$53.1$ m
	RL+LRBS+OA	$0.719698$	$0.0000$	$8.0$ m	$1.437370$	$0.0090$	$42.1$ m	$1.904311$	$0.2255$	$85.7$ m
rdpii	PhyloES	$2.816464$	$0.0000$	$0.2$ m	$5.748068$	$0.0000$	$0.9$ m	$7.784795$	$0.0000$	$2.1$ m
	bspr	$2.816936$	$0.0162$	$0.0$ m	$5.749599$	$0.0266$	$0.0$ m	$7.787445$	$0.0340$	$0.0$ m
	sample-bspr	$2.816805$	$0.0116$	$0.1$ m	$5.749579$	$0.0263$	$0.9$ m	$7.789592$	$0.0616$	$2.1$ m
	fair-bspr	$2.816470$	$0.0002$	$0.0$ m	$5.748206$	$0.0024$	$0.0$ m	$7.785110$	$0.0040$	$0.0$ m
	RL	$2.816648$	$0.0063$	$0.2$ m	$6.827554$	$18.347$	$1.0$ m	$12.972357$	$66.729$	$1.8$ m
	RL+LRBS	$2.816464$	$0.0000$	$4.0$ m	$5.748541$	$0.0081$	$24.4$ m	$10.872700$	$39.711$	$52.6$ m
	RL+LRBS+OA	$2.816465$	$0.0001$	$7.9$ m	$5.748444$	$0.0065$	$42.2$ m	$7.846645$	$0.7880$	$86.3$ m

For the $30$-taxa test cases, we find that switching substitution models only slightly impacts performance, with the RL-based heuristic consistently outperforming both the *bspr* and *sample-bspr* baselines. This highlights the robustness of the trained agent across different evolutionary models. Moreover, when paired with LRBS and online adaptation, the agent nearly eliminates the gap with PhyloES, reaffirming the strength of this combination for smaller problem instances.

As the number of taxa increases, however, the agent’s ability to navigate the larger solution space begins to deteriorate. Specifically, for instances with $100$ taxa, using SPR moves sampled from the learned policy alone can lead to gaps approaching $80\%$, underscoring the difficulty of scaling up without additional search mechanisms. In such scenarios, LRBS becomes essential, enhancing the policy’s performance through broader exploration and online adjustment. For medium-sized problems with $70$ taxa, performance varies notably depending on the training dataset. The policy trained on the *zilla* dataset maintains a gap below $1\%$, indicating strong scaling to moderately larger instances. In contrast, the policy trained on *rdpii* shows much larger gaps, ranging between $15\%$ and $25\%$, suggesting that the nature of the training data significantly influences generalization performance.

Despite these challenges, incorporating LRBS with online exploration mitigates the performance drop in both cases. Additional improvements are observed when online adaptation is included, enabling the RL-based approach to outperform all baselines except for *fair-bspr*. For the most demanding $100$-taxa instances, the substantial distributional shift from training conditions exceeds what LRBS with exploration alone can handle effectively. Nevertheless, as shown in the RL+LRBS+OA rows, augmenting LRBS with online adaptation helps closing the gap to under $1\%$, even though it still falls short of matching *fair-bspr* in some cases. Overall, these results demonstrate that RL-based heuristics can provide a competitive alternative to traditional methods like *bspr* for solving the BMEP. However, achieving strong generalization across both diverse substitution models and increasingly large instances necessitates the integration of online search and adaptation at inference time, components that prove crucial to sustaining performance. While the proposed method generally performs competitively, its performance decreases relative to *fair-bspr* as the instance size increases. For instances with $70$ taxa, the observed gap remains small, suggesting that both the search process and the online adaptation mechanism operate effectively, although additional fine-tuning could further enhance performance. However, for the largest instances with $100$ taxa, the gap becomes more pronounced, indicating that the search space may be too large for the current level of exploration and online adaptation to fully compensate. A potential contributing factor is a bias in the data collected during online adaptation, as rollouts are guided by the pretrained policy rather than by a more exploratory strategy, which may limit the diversity of observed improvements.

### Generalization over different datasets

Ensuring strong performance of ML algorithms on datasets that differ significantly from the training data remains a key challenge for their deployment in real-world applications. In this respect, the previous experiments evaluated the generalization capability of the RL model and the LRBS strategy, but the test instances were drawn from a taxon set that also contained those used during training and validation. As such, some degree of overlap or correlation between training and test distributions likely remained. To further assess the robustness of the model, we designed additional experiments in which the policies were evaluated on completely distinct taxon sets, thereby introducing a substantial distributional shift. The corresponding results are shown in Tables 6 and 7 that report performance when models trained on *rdpii* are applied to *zilla* instances and vice versa. Since earlier findings indicated minimal impact of the substitution model on generalization, these experiments were conducted uniquely under the F81 substitution model.

**Table 6:** Performance evaluation (Obj: average objective value, Gap: average gap, and Time: total time) of model trained on *rdpii* and tested on *zilla* datasets, using F81 substitution model, with $30$, $70,$ and $100$ taxa.

	$N = 30$			$N = 70$			$N = 100$		
Method	Obj.	Gap (%)	Time	Obj.	Gap (%)	Time	Obj.	Gap (%)	Time
PhyloES	$0.717019$	$0.0000$	$0.2$ m	$1.422825$	$0.0000$	$1.1$ m	$1.883850$	$0.0000$	$2.1$ m
bspr	$0.717311$	$0.0398$	$0.0$ m	$1.423435$	$0.0429$	$0.0$ m	$1.885092$	$0.0657$	$0.0$ m
sample-bspr	$0.717217$	$0.0272$	$0.1$ m	$1.423361$	$0.0375$	$0.9$ m	$1.885624$	$0.0938$	$2.1$ m
fair-bspr	$0.717020$	$0.0002$	$0.0$ m	$1.422893$	$0.0049$	$0.0$ m	$1.884011$	$0.0085$	$0.0$ m
RL	$0.717122$	$0.0144$	$0.2$ m	$1.423777$	$0.0671$	$1.0$ m	$1.888508$	$0.2462$	$2.1$ m
RL+LRBS	$0.717019$	$0.0000$	$4.2$ m	$1.422907$	$0.0057$	$24.1$ m	$1.884923$	$0.0571$	$54.2$ m
RL+LRBS+OA	$0.717020$	$0.0002$	$8.0$ m	$1.422903$	$0.0055$	$41.9$ m	$1.885093$	$0.0657$	$84.8$ m

**Table 7:** Performance evaluation (Obj: average objective value, Gap: average gap, and Time: total time) of model trained on *zilla* and tested on *rdpii* datasets, using F81 substitution model, with $30$, $70,$ and $100$ taxa.

	$N = 30$			$N = 70$			$N = 100$		
Method	Obj.	Gap (%)	Time	Obj.	Gap (%)	Time	Obj.	Gap (%)	Time
PhyloES	$2.797912$	$0.0000$	$0.2$ m	$5.711794$	$0.0000$	$0.9$ m	$7.753148$	$0.0000$	$2.2$ m
bspr	$2.798349$	$0.0158$	$0.0$ m	$5.713473$	$0.0294$	$0.0$ m	$7.756417$	$0.0421$	$0.0$ m
sample-bspr	$2.798252$	$0.0122$	$0.1$ m	$5.713241$	$0.0253$	$0.9$ m	$7.757830$	$0.0605$	$2.1$ m
fair-bspr	$2.797924$	$0.0004$	$0.0$ m	$5.711840$	$0.0008$	$0.0$ m	$7.753438$	$0.0037$	$0.0$ m
RL	$3.736825$	$33.669$	$0.2$ m	$8.987075$	$57.462$	$0.9$ m	$12.966783$	$67.363$	$1.8$ m
RL+LRBS	$3.435081$	$22.601$	$3.9$ m	$8.784471$	$53.913$	$22.8$ m	$12.226441$	$57.815$	$52.9$ m
RL+LRBS+OA	$2.798181$	$0.0090$	$8.2$ m	$5.763243$	$0.9024$	$42.8$ m	$7.926805$	$2.2407$	$86.4$ m

In Table 6, we observe that the model trained on *rdpii* generalizes very well to *zilla* instances with a robust performance across most problem sizes. The degradation relative to evaluations on *rdpii* is minimal, and the gap remains low. With online search and adaptation, the neural heuristic matches the strongest baseline on small- and medium-sized instances. For the largest problems, performance aligns with the *bspr* baseline, indicating that challenges in scaling to larger instances persist even when the model adapts very well to new taxon sets.

On the other hand, the reverse setting in Table 7 shows a less favourable picture with the plain RL model struggling to generalize effectively. Even on the smallest $30$-taxa problems, the gap reaches $\sim 30\%$, and the gap increases to $\sim 60\%$ for the largest instances. Moreover, in this difficult scenario, incorporating online search during inference offers only modest gains, suggesting limited adaptability in the face of such a large distribution shift. However, when LRBS is combined with online adaptation, performance improves substantially: for smaller instances, the heuristic outperforms both *bspr* and *sample-bspr*, and for larger instances, the gaps are significantly reduced, although the method still falls behind the strongest baseline (fair-bspr).

These results demonstrate the effectiveness of the proposed approach in both scenarios: when the plain RL model adapts well, the LRBS+OA contributes to further enhancing the performance, while in the more challenging case, it significantly reduces the gap with the state-of-the-art, making our RL-based approach more robust.

## Discussion

Azouri *et al.* [36] have shown that local search through greedy selection, such as in FastME and PhyloES, is not always optimal for phylogenetic inference and that choosing less promising intermediate SPR actions can lead to better performance. RL provides a powerful framework to overcome this limitation and improve the performance of existing heuristics. Yet, it comes with the drawbacks of being a learning-based method, and, as such, scaling and generalization represent key challenges to enable a widespread adoption of RL-based heuristics.

Building upon insights from prior research and the broader neural combinatorial optimization literature, our approach enabled an improvement of the learning methodology. The resulting agent was in fact capable of solving instances with $30$ taxa with very good accuracy, thereby extending the problem size limit of problems addressed with ML approaches in the literature, which was previously capped at 20 [35, 36].

Through extensive experiments, we demonstrated that the learned heuristics, when combined with online search and adaptation strategies, can closely match the performance of state-of-the-art baselines for the BMEP. Notably, this combination proved effective in enhancing the scaling and generalization ability of the trained agents across different substitution models and dataset distributions. We believe this is a crucial property for practical deployment, as it reduces the need for retraining on large, computationally expensive datasets. These results reinforce the conclusion that RL agents trained for SPR move selection in the BMEP can achieve competitive performance, even when evaluated on data that differs significantly from the training distribution. However, the generalization ability of the model is influenced by the choice of training dataset, as demonstrated by the asymmetry between the *zilla*-to-*rdpii* and *rdpii*-to-*zilla* settings. In such cases, search and adaptation mechanisms, such as LRBS, are essential for maintaining strong performance. While in previous results, the use of LRBS alone led to good performance, in this scenario, it can only partially compensate for the RL agent’s poor generalization. Instead, these experiments underscore the pivotal role of inference-time adaptation as LRBS+OA provides an essential and substantial contribution to maintaining performance, also when instances are very different from the ones used in the training phase. Therefore, this represents a promising research direction in scaling RL approaches to larger instances and extending them to broader, more diverse problem settings. In fact, the methodology we employ does not make strong assumptions on the underlying problem, thus only minimal modifications would be required for other problem settings, e.g. maximum-likelihood inference [55].

Our results could be further improved by reducing the computational costs as well as the memory usage associated with the RL-based method and LRBS, since currently their implementation largely relies on Python while other baseline methods leverage highly optimized implementations in C++. Much of this overhead stems from the feature extraction process, which plays a central role in guiding the policy’s decisions. However, this stage is highly suitable for parallelization and optimization, and with the right infrastructure, the runtime could be significantly reduced. We did not pursue further optimizations here, as our aim was to show that RL heuristics can outperform greedy strategies and to study their generalization. Further speedups could be explored in future work that aims to integrate these methods into more advanced pipelines, which have the potential to set a new state-of-the-art, and this is a promising direction that we leave for future work.

## Conclusion

In this work, we proposed an improved RL framework for addressing phylogenetic inference in the context of the BMEP using RL-based heuristics. By integrating online search and adaptation mechanisms, we showed that RL approaches can efficiently scale to larger instances and generalize over different substitution models and taxon sets, compared with those used for training. Thus, our approach makes a step toward a key requirement for making these techniques suitable for tackling large phylogenetic inference problems.

**Key Points**

- Reconstructing phylogenetic trees from molecular data under the BMEP requires navigating complex phylogenetic tree spaces. Traditional heuristic methods often rely on local search operators and greedy selection, which have been proven to be suboptimal.
- We introduce a novel framework to tackle the BMEP as an RL problem, enabling an agent to learn effective strategies for exploring tree neighbourhoods.
- We used the learned policy to guide a search process designed to improve its performance, scalability and generalization, tasks which were not addressed in previous RL approaches for phylogenetic inference.
- Our approach outperforms greedy heuristics and achieves results comparable to state-of-the-art methods for the BMEP.
- Our model, although trained on small instances and on a single substitution model, maintains competitive performance across different substitution models, varying dataset sizes, and instances with up to 100 taxa, showing strong scalability and generalization capabilities.

## Data Availability

The code for the approach presented in this paper, as well as the datasets used for the experiments, are available on GitHub (https://github.com/federico-camerota/bmepRL).
